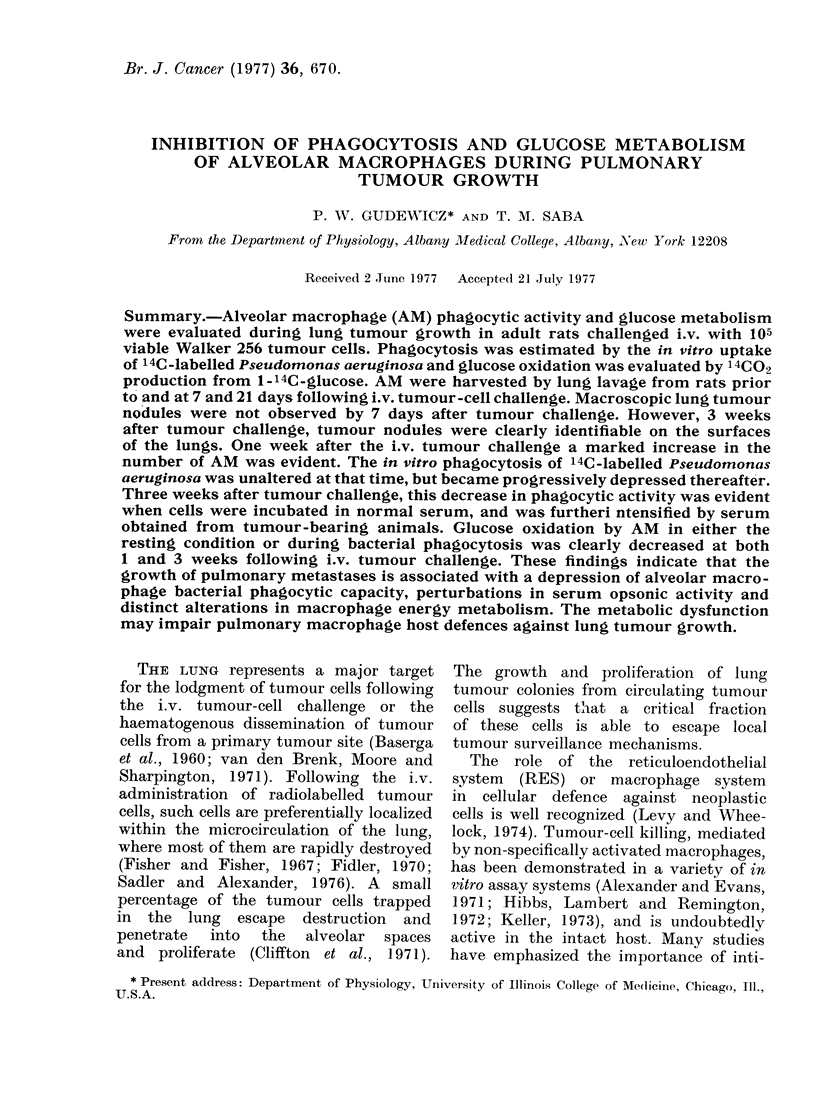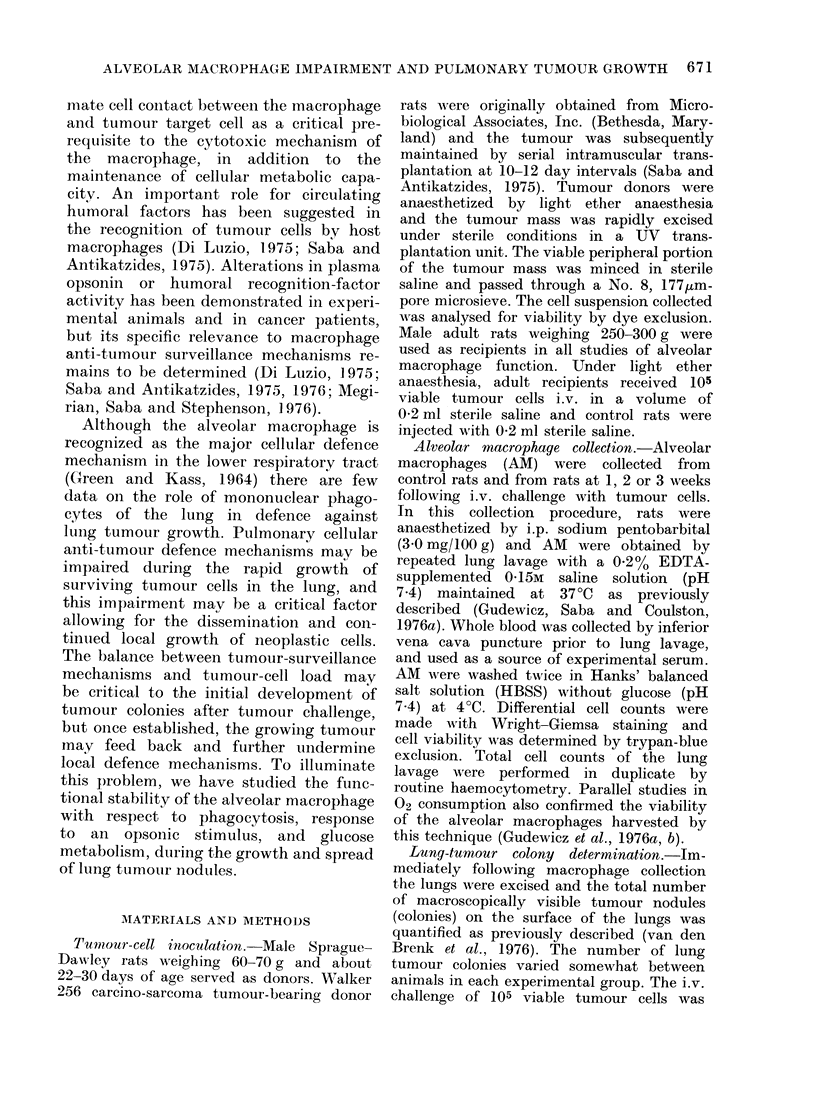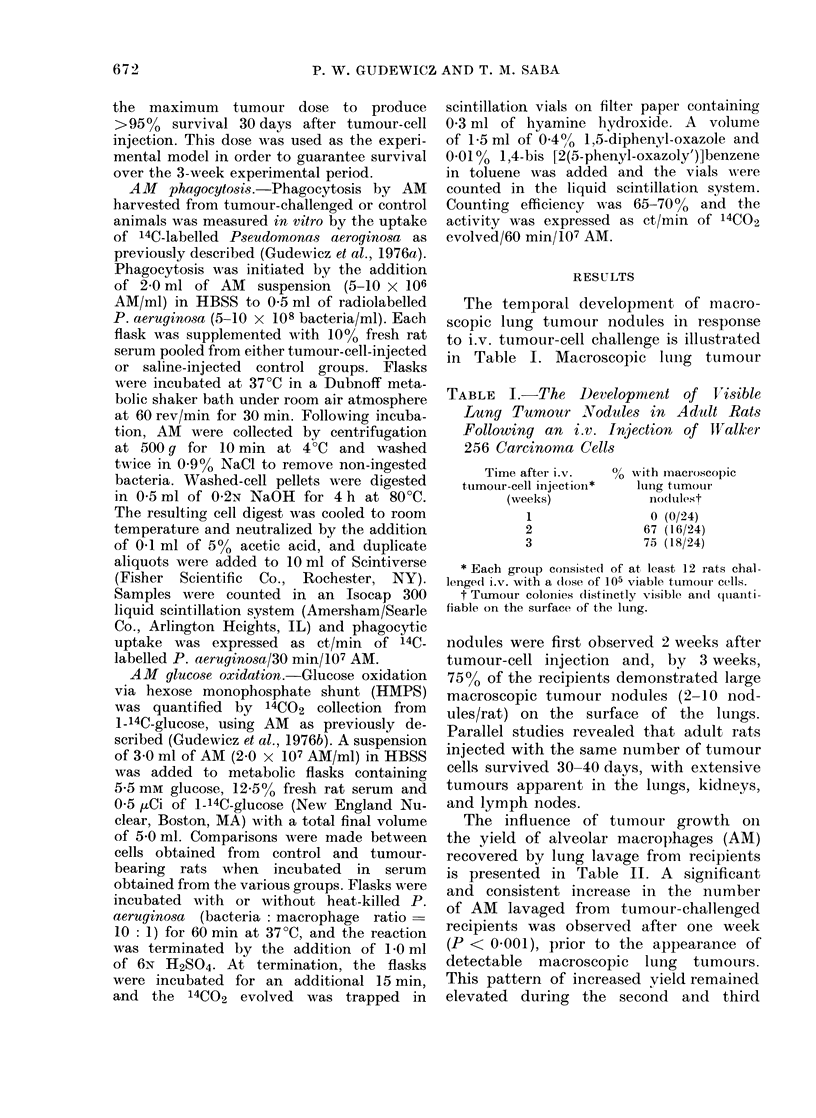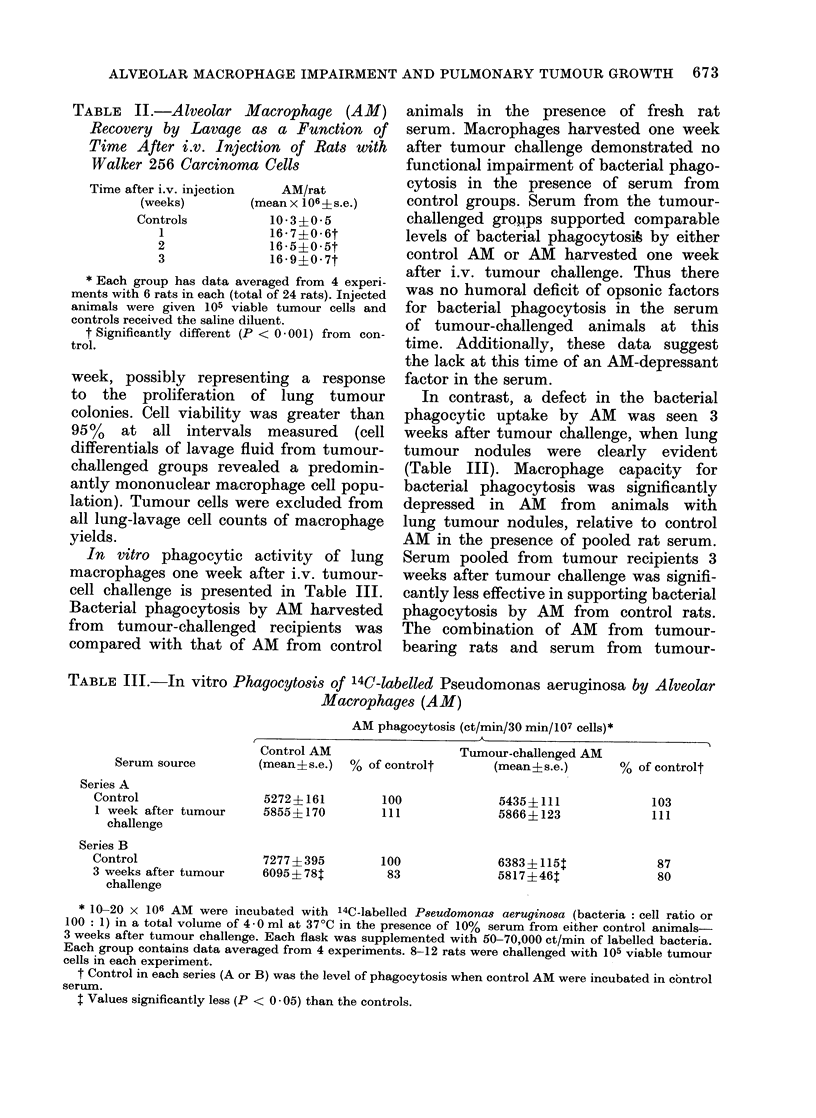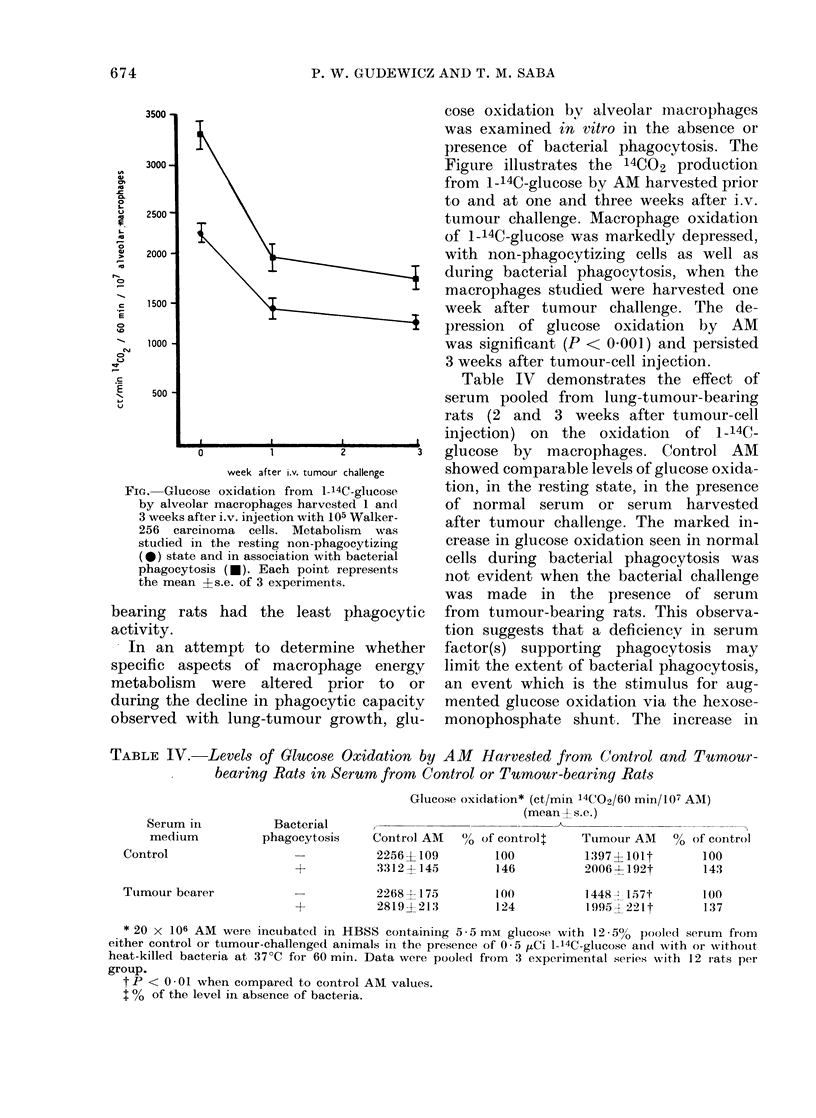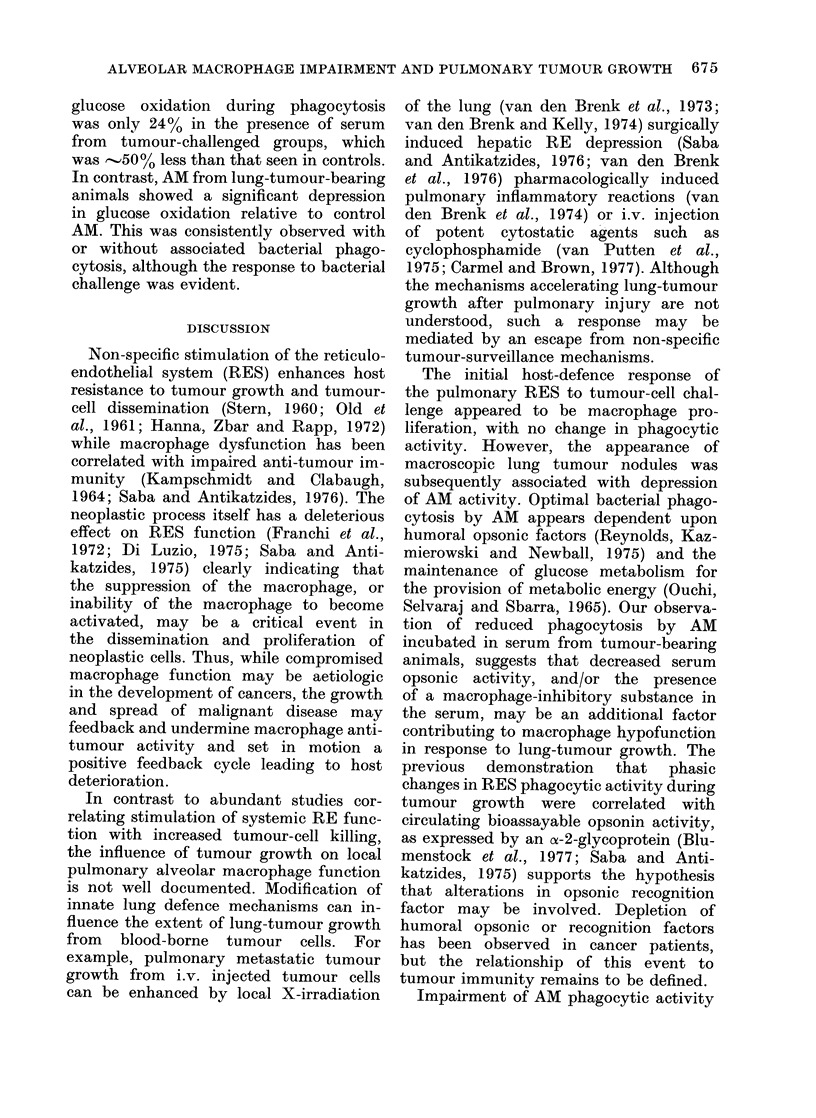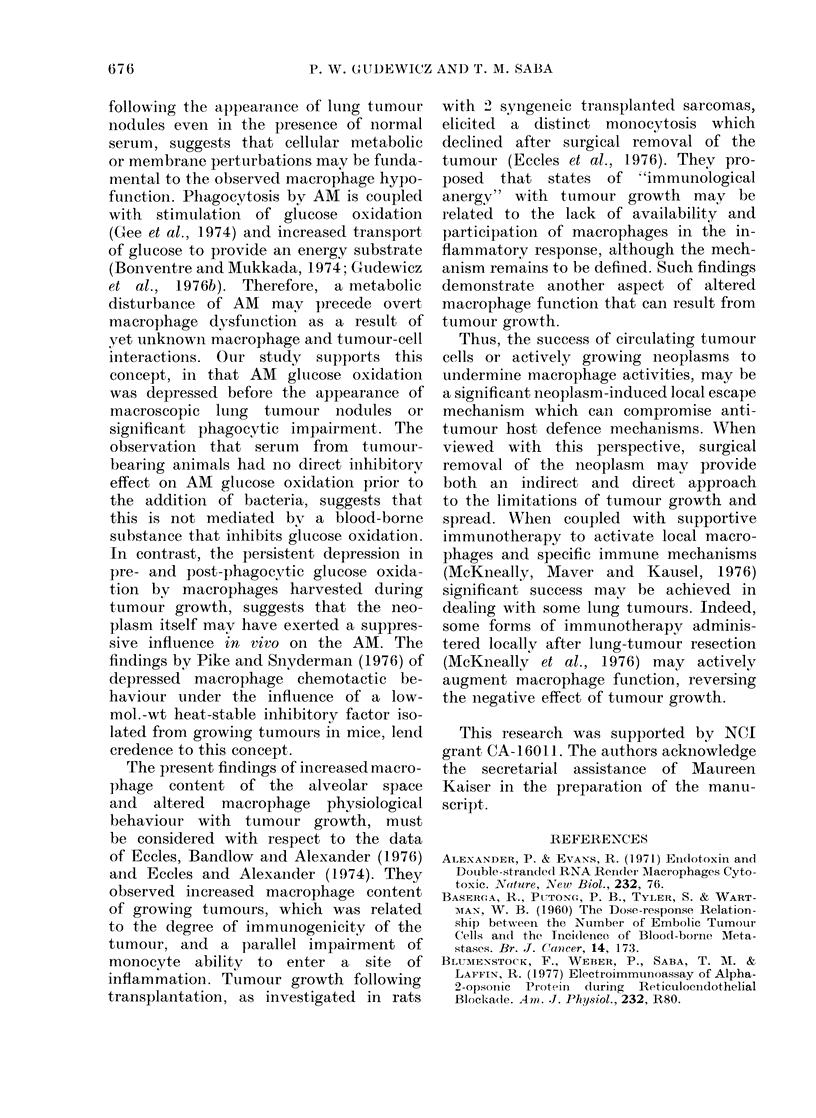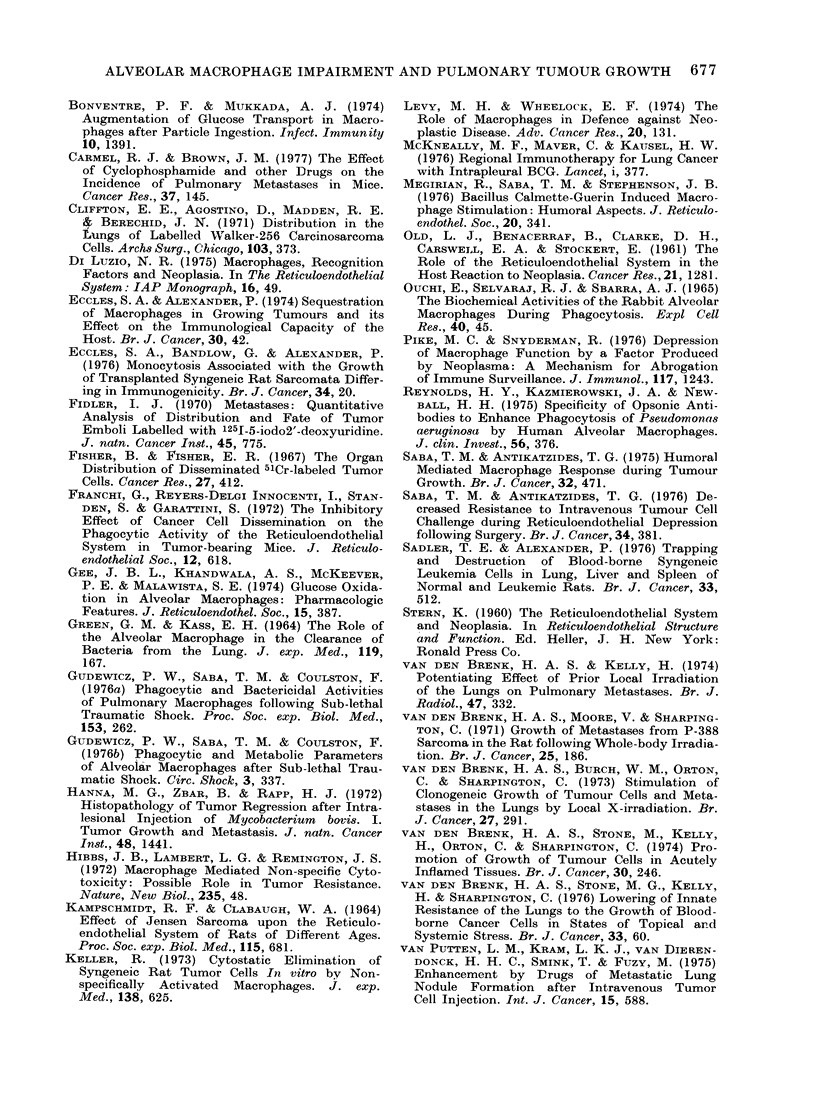# Inhibition of Phagocytosis and Glucose Metabolism of Alveolar Macrophages during Pulmonary Tumour Growth

**DOI:** 10.1038/bjc.1977.249

**Published:** 1977-12

**Authors:** P. W. Gudewicz, T. M. Saba

## Abstract

Alveolar macrophage (AM) phagocytic activity and glucose metabolism were evaluated during lung tumour growth in adult rats challenged i.v. with 10^5^ viable Walker 256 tumour cells. Phagocytosis was estimated by the *in vitro* uptake of ^14^C-labelled *Pseudomonas aeruginosa* and glucose oxidation was evaluated by ^14^CO_2_ production from 1-^14^C-glucose. AM were harvested by lung lavage from rats prior to and at 7 and 21 days following i.v. tumour-cell challenge. Macroscopic lung tumour nodules were not observed by 7 days after tumour challenge. However, 3 weeks after tumour challenge, tumour nodules were clearly identifiable on the surfaces of the lungs. One week after the i.v. tumour challenge a marked increase in the number of AM was evident. The *in vitro* phagocytosis of ^14^C-labelled *Pseudomonas aeruginosa* was unaltered at that time, but became progressively depressed thereafter. Three weeks after tumour challenge, this decrease in phagocytic activity was evident when cells were incubated in normal serum, and was furtheri ntensified by serum obtained from tumour-bearing animals. Glucose oxidation by AM in either the resting condition or during bacterial phagocytosis was clearly decreased at both 1 and 3 weeks following i.v. tumour challenge. These findings indicate that the growth of pulmonary metastases is associated with a depression of alveolar macrophage bacterial phagocytic capacity, perturbations in serum opsonic activity and distinct alterations in macrophage energy metabolism. The metabolic dysfunction may impair pulmonary macrophage host defences against lung tumour growth.


*Br. J. Cancer* (1977) **36**, 670.

### INHIBITION OF PHAGOCYTOSIS AND GLUCOSE METABOLISM OF ALVEOLAR MACROPHAGES DURING PULMONARY TUMOUR GROWTH

P. W. GUDEWICZ* AND T. M. SABA

*From the Department of Physiology, Albany Medical College, Albany, New York* 12208

Received 2 June 1977 Accepted 21 July 1977

**Summary.—Alveolar macrophage (AM) phagocytic activity and glucose metabolism were evaluated during lung tumour growth in adult rats challenged i.v. with $10^5$ viable Walker 256 tumour cells. Phagocytosis was estimated by the *in vitro* uptake of $^{14}C$-labelled *Pseudomonas aeruginosa* and glucose oxidation was evaluated by $^{14}CO_2$ production from 1-$^{14}C$-glucose. AM were harvested by lung lavage from rats prior to and at 7 and 21 days following i.v. tumour-cell challenge. Macroscopic lung tumour nodules were not observed by 7 days after tumour challenge. However, 3 weeks after tumour challenge, tumour nodules were clearly identifiable on the surfaces of the lungs. One week after the i.v. tumour challenge a marked increase in the number of AM was evident. The *in vitro* phagocytosis of $^{14}C$-labelled *Pseudomonas aeruginosa* was unaltered at that time, but became progressively depressed thereafter. Three weeks after tumour challenge, this decrease in phagocytic activity was evident when cells were incubated in normal serum, and was furtheri ntensified by serum obtained from tumour-bearing animals. Glucose oxidation by AM in either the resting condition or during bacterial phagocytosis was clearly decreased at both 1 and 3 weeks following i.v. tumour challenge. These findings indicate that the growth of pulmonary metastases is associated with a depression of alveolar macrophage bacterial phagocytic capacity, perturbations in serum opsonic activity and distinct alterations in macrophage energy metabolism. The metabolic dysfunction may impair pulmonary macrophage host defences against lung tumour growth.**

THE LUNG represents a major target for the lodgment of tumour cells following the i.v. tumour-cell challenge or the haematogenous dissemination of tumour cells from a primary tumour site (Baserga *et al.*, 1960; van den Brenk, Moore and Sharpington, 1971). Following the i.v. administration of radiolabelled tumour cells, such cells are preferentially localized within the microcirculation of the lung, where most of them are rapidly destroyed (Fisher and Fisher, 1967; Fidler, 1970; Sadler and Alexander, 1976). A small percentage of the tumour cells trapped in the lung escape destruction and penetrate into the alveolar spaces and proliferate (Cliffton *et al.*, 1971). The growth and proliferation of lung tumour colonies from circulating tumour cells suggests that a critical fraction of these cells is able to escape local tumour surveillance mechanisms.

The role of the reticuloendothelial system (RES) or macrophage system in cellular defence against neoplastic cells is well recognized (Levy and Wheelock, 1974). Tumour-cell killing, mediated by non-specifically activated macrophages, has been demonstrated in a variety of *in vitro* assay systems (Alexander and Evans, 1971; Hibbs, Lambert and Remington, 1972; Keller, 1973), and is undoubtedly active in the intact host. Many studies have emphasized the importance of inti-

* Present address: Department of Physiology, University of Illinois College of Medicine, Chicago, Ill., U.S.A.

mate cell contact between the macrophage and tumour target cell as a critical prerequisite to the cytotoxic mechanism of the macrophage, in addition to the maintenance of cellular metabolic capacity. An important role for circulating humoral factors has been suggested in the recognition of tumour cells by host macrophages (Di Luzio, 1975; Saba and Antikatzides, 1975). Alterations in plasma opsonin or humoral recognition-factor activity has been demonstrated in experimental animals and in cancer patients, but its specific relevance to macrophage anti-tumour surveillance mechanisms remains to be determined (Di Luzio, 1975; Saba and Antikatzides, 1975, 1976; Megirian, Saba and Stephenson, 1976).

Although the alveolar macrophage is recognized as the major cellular defence mechanism in the lower respiratory tract (Green and Kass, 1964) there are few data on the role of mononuclear phagocytes of the lung in defence against lung tumour growth. Pulmonary cellular anti-tumour defence mechanisms may be impaired during the rapid growth of surviving tumour cells in the lung, and this impairment may be a critical factor allowing for the dissemination and continued local growth of neoplastic cells. The balance between tumour-surveillance mechanisms and tumour-cell load may be critical to the initial development of tumour colonies after tumour challenge, but once established, the growing tumour may feed back and further undermine local defence mechanisms. To illuminate this problem, we have studied the functional stability of the alveolar macrophage with respect to phagocytosis, response to an opsonic stimulus, and glucose metabolism, during the growth and spread of lung tumour nodules.

### MATERIALS AND METHODS

*Tumour-cell inoculation.*—Male Sprague–Dawley rats weighing 60–70 g and about 22–30 days of age served as donors. Walker 256 carcino-sarcoma tumour-bearing donor rats were originally obtained from Microbiological Associates, Inc. (Bethesda, Maryland) and the tumour was subsequently maintained by serial intramuscular transplantation at 10–12 day intervals (Saba and Antikatzides, 1975). Tumour donors were anaesthetized by light ether anaesthesia and the tumour mass was rapidly excised under sterile conditions in a UV transplantation unit. The viable peripheral portion of the tumour mass was minced in sterile saline and passed through a No. 8, 177$\mu$m-pore microsieve. The cell suspension collected was analysed for viability by dye exclusion. Male adult rats weighing 250–300 g were used as recipients in all studies of alveolar macrophage function. Under light ether anaesthesia, adult recipients received $10^5$ viable tumour cells i.v. in a volume of 0·2 ml sterile saline and control rats were injected with 0·2 ml sterile saline.

*Alveolar macrophage collection.*—Alveolar macrophages (AM) were collected from control rats and from rats at 1, 2 or 3 weeks following i.v. challenge with tumour cells. In this collection procedure, rats were anaesthetized by i.p. sodium pentobarbital (3·0 mg/100 g) and AM were obtained by repeated lung lavage with a 0·2% EDTA-supplemented 0·15M saline solution (pH 7·4) maintained at 37°C as previously described (Gudewicz, Saba and Coulston, 1976*a*). Whole blood was collected by inferior vena cava puncture prior to lung lavage, and used as a source of experimental serum. AM were washed twice in Hanks' balanced salt solution (HBSS) without glucose (pH 7·4) at 4°C. Differential cell counts were made with Wright–Giemsa staining and cell viability was determined by trypan-blue exclusion. Total cell counts of the lung lavage were performed in duplicate by routine haemocytometry. Parallel studies in $O_2$ consumption also confirmed the viability of the alveolar macrophages harvested by this technique (Gudewicz *et al.*, 1976*a*, *b*).

*Lung-tumour colony determination.*—Immediately following macrophage collection the lungs were excised and the total number of macroscopically visible tumour nodules (colonies) on the surface of the lungs was quantified as previously described (van den Brenk *et al.*, 1976). The number of lung tumour colonies varied somewhat between animals in each experimental group. The i.v. challenge of $10^5$ viable tumour cells was the maximum tumour dose to produce >95% survival 30 days after tumour-cell injection. This dose was used as the experimental model in order to guarantee survival over the 3-week experimental period.

*AM phagocytosis.*—Phagocytosis by AM harvested from tumour-challenged or control animals was measured *in vitro* by the uptake of $^{14}C$-labelled *Pseudomonas aeroginosa* as previously described (Gudewicz *et al.*, 1976*a*). Phagocytosis was initiated by the addition of 2·0 ml of AM suspension (5–10 × $10^6$ AM/ml) in HBSS to 0·5 ml of radiolabelled *P. aeruginosa* (5–10 × $10^8$ bacteria/ml). Each flask was supplemented with 10% fresh rat serum pooled from either tumour-cell-injected or saline-injected control groups. Flasks were incubated at 37°C in a Dubnoff metabolic shaker bath under room air atmosphere at 60 rev/min for 30 min. Following incubation, AM were collected by centrifugation at 500 *g* for 10 min at 4°C and washed twice in 0·9% NaCl to remove non-ingested bacteria. Washed-cell pellets were digested in 0·5 ml of 0·2N NaOH for 4 h at 80°C. The resulting cell digest was cooled to room temperature and neutralized by the addition of 0·1 ml of 5% acetic acid, and duplicate aliquots were added to 10 ml of Scintiverse (Fisher Scientific Co., Rochester, NY). Samples were counted in an Isocap 300 liquid scintillation system (Amersham/Searle Co., Arlington Heights, IL) and phagocytic uptake was expressed as ct/min of $^{14}C$-labelled *P. aeruginosa*/30 min/$10^7$ AM.

*AM glucose oxidation.*—Glucose oxidation via hexose monophosphate shunt (HMPS) was quantified by $^{14}CO_2$ collection from 1-$^{14}C$-glucose, using AM as previously described (Gudewicz *et al.*, 1976*b*). A suspension of 3·0 ml of AM (2·0 × $10^7$ AM/ml) in HBSS was added to metabolic flasks containing 5·5 mM glucose, 12·5% fresh rat serum and 0·5 $\mu$Ci of 1-$^{14}C$-glucose (New England Nuclear, Boston, MA) with a total final volume of 5·0 ml. Comparisons were made between cells obtained from control and tumour-bearing rats when incubated in serum obtained from the various groups. Flasks were incubated with or without heat-killed *P. aeruginosa* (bacteria : macrophage ratio = 10 : 1) for 60 min at 37°C, and the reaction was terminated by the addition of 1·0 ml of 6N $H_2SO_4$. At termination, the flasks were incubated for an additional 15 min, and the $^{14}CO_2$ evolved was trapped in scintillation vials on filter paper containing 0·3 ml of hyamine hydroxide. A volume of 1·5 ml of 0·4% 1,5-diphenyl-oxazole and 0·01% 1,4-bis [2(5-phenyl-oxazoly')]benzene in toluene was added and the vials were counted in the liquid scintillation system. Counting efficiency was 65–70% and the activity was expressed as ct/min of $^{14}CO_2$ evolved/60 min/$10^7$ AM.

### RESULTS

The temporal development of macroscopic lung tumour nodules in response to i.v. tumour-cell challenge is illustrated in Table I. Macroscopic lung tumour nodules were first observed 2 weeks after tumour-cell injection and, by 3 weeks, 75% of the recipients demonstrated large macroscopic tumour nodules (2–10 nodules/rat) on the surface of the lungs. Parallel studies revealed that adult rats injected with the same number of tumour cells survived 30–40 days, with extensive tumours apparent in the lungs, kidneys, and lymph nodes.

TABLE I.—*The Development of Visible Lung Tumour Nodules in Adult Rats Following an i.v. Injection of Walker 256 Carcinoma Cells*

| Time after i.v. tumour-cell injection* (weeks) | % with macroscopic lung tumour nodules† |
|---|---|
| 1 | 0 (0/24) |
| 2 | 67 (16/24) |
| 3 | 75 (18/24) |

* Each group consisted of at least 12 rats challenged i.v. with a dose of $10^5$ viable tumour cells.

† Tumour colonies distinctly visible and quantifiable on the surface of the lung.

The influence of tumour growth on the yield of alveolar macrophages (AM) recovered by lung lavage from recipients is presented in Table II. A significant and consistent increase in the number of AM lavaged from tumour-challenged recipients was observed after one week ($P < 0{\cdot}001$), prior to the appearance of detectable macroscopic lung tumours. This pattern of increased yield remained elevated during the second and third

TABLE II.—*Alveolar Macrophage (AM) Recovery by Lavage as a Function of Time After i.v. Injection of Rats with Walker 256 Carcinoma Cells*

| Time after i.v. injection (weeks) | AM/rat (mean $\times 10^6 \pm$ s.e.) |
|---|---|
| Controls | $10 \cdot 3 \pm 0 \cdot 5$ |
| 1 | $16 \cdot 7 \pm 0 \cdot 6$† |
| 2 | $16 \cdot 5 \pm 0 \cdot 5$† |
| 3 | $16 \cdot 9 \pm 0 \cdot 7$† |

* Each group has data averaged from 4 experiments with 6 rats in each (total of 24 rats). Injected animals were given $10^5$ viable tumour cells and controls received the saline diluent.

† Significantly different ($P < 0 \cdot 001$) from control.

week, possibly representing a response to the proliferation of lung tumour colonies. Cell viability was greater than 95% at all intervals measured (cell differentials of lavage fluid from tumour-challenged groups revealed a predominantly mononuclear macrophage cell population). Tumour cells were excluded from all lung-lavage cell counts of macrophage yields.

*In vitro* phagocytic activity of lung macrophages one week after i.v. tumour-cell challenge is presented in Table III. Bacterial phagocytosis by AM harvested from tumour-challenged recipients was compared with that of AM from control animals in the presence of fresh rat serum. Macrophages harvested one week after tumour challenge demonstrated no functional impairment of bacterial phagocytosis in the presence of serum from control groups. Serum from the tumour-challenged groups supported comparable levels of bacterial phagocytosis by either control AM or AM harvested one week after i.v. tumour challenge. Thus there was no humoral deficit of opsonic factors for bacterial phagocytosis in the serum of tumour-challenged animals at this time. Additionally, these data suggest the lack at this time of an AM-depressant factor in the serum.

In contrast, a defect in the bacterial phagocytic uptake by AM was seen 3 weeks after tumour challenge, when lung tumour nodules were clearly evident (Table III). Macrophage capacity for bacterial phagocytosis was significantly depressed in AM from animals with lung tumour nodules, relative to control AM in the presence of pooled rat serum. Serum pooled from tumour recipients 3 weeks after tumour challenge was significantly less effective in supporting bacterial phagocytosis by AM from control rats. The combination of AM from tumour-bearing rats and serum from tumour-

TABLE III.—In vitro *Phagocytosis of $^{14}$C-labelled* Pseudomonas aeruginosa *by Alveolar Macrophages (AM)*

| | AM phagocytosis (ct/min/30 min/$10^7$ cells)* | | | |
|---|---|---|---|---|
| Serum source | Control AM (mean $\pm$ s.e.) | % of control† | Tumour-challenged AM (mean $\pm$ s.e.) | % of control† |
| Series A | | | | |
| Control | $5272 \pm 161$ | 100 | $5435 \pm 111$ | 103 |
| 1 week after tumour challenge | $5855 \pm 170$ | 111 | $5866 \pm 123$ | 111 |
| Series B | | | | |
| Control | $7277 \pm 395$ | 100 | $6383 \pm 115$‡ | 87 |
| 3 weeks after tumour challenge | $6095 \pm 78$‡ | 83 | $5817 \pm 46$‡ | 80 |

* 10–20 × $10^6$ AM were incubated with $^{14}$C-labelled *Pseudomonas aeruginosa* (bacteria : cell ratio or 100 : 1) in a total volume of $4 \cdot 0$ ml at 37°C in the presence of 10% serum from either control animals—3 weeks after tumour challenge. Each flask was supplemented with 50–70,000 ct/min of labelled bacteria. Each group contains data averaged from 4 experiments. 8–12 rats were challenged with $10^5$ viable tumour cells in each experiment.

† Control in each series (A or B) was the level of phagocytosis when control AM were incubated in control serum.

‡ Values significantly less ($P < 0 \cdot 05$) than the controls.

FIG.—Glucose oxidation from 1-$^{14}$C-glucose by alveolar macrophages harvested 1 and 3 weeks after i.v. injection with $10^5$ Walker-256 carcinoma cells. Metabolism was studied in the resting non-phagocytizing (●) state and in association with bacterial phagocytosis (■). Each point represents the mean ±s.e. of 3 experiments.

bearing rats had the least phagocytic activity.

In an attempt to determine whether specific aspects of macrophage energy metabolism were altered prior to or during the decline in phagocytic capacity observed with lung-tumour growth, glucose oxidation by alveolar macrophages was examined *in vitro* in the absence or presence of bacterial phagocytosis. The Figure illustrates the $^{14}CO_2$ production from 1-$^{14}$C-glucose by AM harvested prior to and at one and three weeks after i.v. tumour challenge. Macrophage oxidation of 1-$^{14}$C-glucose was markedly depressed, with non-phagocytizing cells as well as during bacterial phagocytosis, when the macrophages studied were harvested one week after tumour challenge. The depression of glucose oxidation by AM was significant ($P < 0{\cdot}001$) and persisted 3 weeks after tumour-cell injection.

Table IV demonstrates the effect of serum pooled from lung-tumour-bearing rats (2 and 3 weeks after tumour-cell injection) on the oxidation of 1-$^{14}$C-glucose by macrophages. Control AM showed comparable levels of glucose oxidation, in the resting state, in the presence of normal serum or serum harvested after tumour challenge. The marked increase in glucose oxidation seen in normal cells during bacterial phagocytosis was not evident when the bacterial challenge was made in the presence of serum from tumour-bearing rats. This observation suggests that a deficiency in serum factor(s) supporting phagocytosis may limit the extent of bacterial phagocytosis, an event which is the stimulus for augmented glucose oxidation via the hexose-monophosphate shunt. The increase in

TABLE IV.—*Levels of Glucose Oxidation by AM Harvested from Control and Tumour-bearing Rats in Serum from Control or Tumour-bearing Rats*

| Serum in medium | Bacterial phagocytosis | Glucose oxidation* (ct/min $^{14}CO_2$/60 min/$10^7$ AM) (mean ± s.e.) Control AM | % of control‡ | Tumour AM | % of control |
|---|---|---|---|---|---|
| Control | − | 2256 ± 109 | 100 | 1397 ± 101† | 100 |
| | + | 3312 ± 145 | 146 | 2006 ± 192† | 143 |
| Tumour bearer | − | 2268 ± 175 | 100 | 1448 ± 157† | 100 |
| | + | 2819 ± 213 | 124 | 1995 ± 221† | 137 |

* 20 × $10^6$ AM were incubated in HBSS containing 5·5 mM glucose with 12·5% pooled serum from either control or tumour-challenged animals in the presence of 0·5 μCi 1-$^{14}$C-glucose and with or without heat-killed bacteria at 37°C for 60 min. Data were pooled from 3 experimental series with 12 rats per group.

† $P < 0{\cdot}01$ when compared to control AM values.

‡ % of the level in absence of bacteria.

glucose oxidation during phagocytosis was only 24% in the presence of serum from tumour-challenged groups, which was ~50% less than that seen in controls. In contrast, AM from lung-tumour-bearing animals showed a significant depression in glucose oxidation relative to control AM. This was consistently observed with or without associated bacterial phagocytosis, although the response to bacterial challenge was evident.

### DISCUSSION

Non-specific stimulation of the reticuloendothelial system (RES) enhances host resistance to tumour growth and tumour-cell dissemination (Stern, 1960; Old *et al.*, 1961; Hanna, Zbar and Rapp, 1972) while macrophage dysfunction has been correlated with impaired anti-tumour immunity (Kampschmidt and Clabaugh, 1964; Saba and Antikatzides, 1976). The neoplastic process itself has a deleterious effect on RES function (Franchi *et al.*, 1972; Di Luzio, 1975; Saba and Antikatzides, 1975) clearly indicating that the suppression of the macrophage, or inability of the macrophage to become activated, may be a critical event in the dissemination and proliferation of neoplastic cells. Thus, while compromised macrophage function may be aetiologic in the development of cancers, the growth and spread of malignant disease may feedback and undermine macrophage anti-tumour activity and set in motion a positive feedback cycle leading to host deterioration.

In contrast to abundant studies correlating stimulation of systemic RE function with increased tumour-cell killing, the influence of tumour growth on local pulmonary alveolar macrophage function is not well documented. Modification of innate lung defence mechanisms can influence the extent of lung-tumour growth from blood-borne tumour cells. For example, pulmonary metastatic tumour growth from i.v. injected tumour cells can be enhanced by local X-irradiation of the lung (van den Brenk *et al.*, 1973; van den Brenk and Kelly, 1974) surgically induced hepatic RE depression (Saba and Antikatzides, 1976; van den Brenk *et al.*, 1976) pharmacologically induced pulmonary inflammatory reactions (van den Brenk *et al.*, 1974) or i.v. injection of potent cytostatic agents such as cyclophosphamide (van Putten *et al.*, 1975; Carmel and Brown, 1977). Although the mechanisms accelerating lung-tumour growth after pulmonary injury are not understood, such a response may be mediated by an escape from non-specific tumour-surveillance mechanisms.

The initial host-defence response of the pulmonary RES to tumour-cell challenge appeared to be macrophage proliferation, with no change in phagocytic activity. However, the appearance of macroscopic lung tumour nodules was subsequently associated with depression of AM activity. Optimal bacterial phagocytosis by AM appears dependent upon humoral opsonic factors (Reynolds, Kazmierowski and Newball, 1975) and the maintenance of glucose metabolism for the provision of metabolic energy (Ouchi, Selvaraj and Sbarra, 1965). Our observation of reduced phagocytosis by AM incubated in serum from tumour-bearing animals, suggests that decreased serum opsonic activity, and/or the presence of a macrophage-inhibitory substance in the serum, may be an additional factor contributing to macrophage hypofunction in response to lung-tumour growth. The previous demonstration that phasic changes in RES phagocytic activity during tumour growth were correlated with circulating bioassayable opsonin activity, as expressed by an $\alpha$-2-glycoprotein (Blumenstock *et al.*, 1977; Saba and Antikatzides, 1975) supports the hypothesis that alterations in opsonic recognition factor may be involved. Depletion of humoral opsonic or recognition factors has been observed in cancer patients, but the relationship of this event to tumour immunity remains to be defined.

Impairment of AM phagocytic activity following the appearance of lung tumour nodules even in the presence of normal serum, suggests that cellular metabolic or membrane perturbations may be fundamental to the observed macrophage hypofunction. Phagocytosis by AM is coupled with stimulation of glucose oxidation (Gee *et al.*, 1974) and increased transport of glucose to provide an energy substrate (Bonventre and Mukkada, 1974; Gudewicz *et al.*, 1976*b*). Therefore, a metabolic disturbance of AM may precede overt macrophage dysfunction as a result of yet unknown macrophage and tumour-cell interactions. Our study supports this concept, in that AM glucose oxidation was depressed before the appearance of macroscopic lung tumour nodules or significant phagocytic impairment. The observation that serum from tumour-bearing animals had no direct inhibitory effect on AM glucose oxidation prior to the addition of bacteria, suggests that this is not mediated by a blood-borne substance that inhibits glucose oxidation. In contrast, the persistent depression in pre- and post-phagocytic glucose oxidation by macrophages harvested during tumour growth, suggests that the neoplasm itself may have exerted a suppressive influence *in vivo* on the AM. The findings by Pike and Snyderman (1976) of depressed macrophage chemotactic behaviour under the influence of a low-mol.-wt heat-stable inhibitory factor isolated from growing tumours in mice, lend credence to this concept.

The present findings of increased macrophage content of the alveolar space and altered macrophage physiological behaviour with tumour growth, must be considered with respect to the data of Eccles, Bandlow and Alexander (1976) and Eccles and Alexander (1974). They observed increased macrophage content of growing tumours, which was related to the degree of immunogenicity of the tumour, and a parallel impairment of monocyte ability to enter a site of inflammation. Tumour growth following transplantation, as investigated in rats with 2 syngeneic transplanted sarcomas, elicited a distinct monocytosis which declined after surgical removal of the tumour (Eccles *et al.*, 1976). They proposed that states of "immunological anergy" with tumour growth may be related to the lack of availability and participation of macrophages in the inflammatory response, although the mechanism remains to be defined. Such findings demonstrate another aspect of altered macrophage function that can result from tumour growth.

Thus, the success of circulating tumour cells or actively growing neoplasms to undermine macrophage activities, may be a significant neoplasm-induced local escape mechanism which can compromise anti-tumour host defence mechanisms. When viewed with this perspective, surgical removal of the neoplasm may provide both an indirect and direct approach to the limitations of tumour growth and spread. When coupled with supportive immunotherapy to activate local macrophages and specific immune mechanisms (McKneally, Maver and Kausel, 1976) significant success may be achieved in dealing with some lung tumours. Indeed, some forms of immunotherapy administered locally after lung-tumour resection (McKneally *et al.*, 1976) may actively augment macrophage function, reversing the negative effect of tumour growth.

This research was supported by NCI grant CA-16011. The authors acknowledge the secretarial assistance of Maureen Kaiser in the preparation of the manuscript.

### REFERENCES

ALEXANDER, P. & EVANS, R. (1971) Endotoxin and Double-stranded RNA Render Macrophages Cytotoxic. *Nature, New Biol.*, **232**, 76.

BASERGA, R., PUTONG, P. B., TYLER, S. & WARTMAN, W. B. (1960) The Dose-response Relationship between the Number of Embolic Tumour Cells and the Incidence of Blood-borne Metastases. *Br. J. Cancer*, **14**, 173.

BLUMENSTOCK, F., WEBER, P., SABA, T. M. & LAFFIN, R. (1977) Electroimmunoassay of Alpha-2-opsonic Protein during Reticuloendothelial Blockade. *Am. J. Physiol.*, **232**, R80.

BONVENTRE, P. F. & MUKKADA, A. J. (1974) Augmentation of Glucose Transport in Macrophages after Particle Ingestion. *Infect. Immunity* **10**, 1391.

CARMEL, R. J. & BROWN, J. M. (1977) The Effect of Cyclophosphamide and other Drugs on the Incidence of Pulmonary Metastases in Mice. *Cancer Res.*, **37**, 145.

CLIFFTON, E. E., AGOSTINO, D., MADDEN, R. E. & BERECHID, J. N. (1971) Distribution in the Lungs of Labelled Walker-256 Carcinosarcoma Cells. *Archs Surg., Chicago*, **103**, 373.

DI LUZIO, N. R. (1975) Macrophages, Recognition Factors and Neoplasia. In *The Reticuloendothelial System: IAP Monograph*, **16**, 49.

ECCLES, S. A. & ALEXANDER, P. (1974) Sequestration of Macrophages in Growing Tumours and its Effect on the Immunological Capacity of the Host. *Br. J. Cancer*, **30**, 42.

ECCLES, S. A., BANDLOW, G. & ALEXANDER, P. (1976) Monocytosis Associated with the Growth of Transplanted Syngeneic Rat Sarcomata Differing in Immunogenicity. *Br. J. Cancer*, **34**, 20.

FIDLER, I. J. (1970) Metastases: Quantitative Analysis of Distribution and Fate of Tumor Emboli Labelled with $^{125}$I-5-iodo2′-deoxyuridine. *J. natn. Cancer Inst.*, **45**, 775.

FISHER, B. & FISHER, E. R. (1967) The Organ Distribution of Disseminated $^{51}$Cr-labeled Tumor Cells. *Cancer Res.*, **27**, 412.

FRANCHI, G., REYERS-DELGI INNOCENTI, I., STANDEN, S. & GARATTINI, S. (1972) The Inhibitory Effect of Cancer Cell Dissemination on the Phagocytic Activity of the Reticuloendothelial System in Tumor-bearing Mice. *J. Reticuloendothelial Soc.*, **12**, 618.

GEE, J. B. L., KHANDWALA, A. S., MCKEEVER, P. E. & MALAWISTA, S. E. (1974) Glucose Oxidation in Alveolar Macrophages: Pharmacologic Features. *J. Reticuloendothel. Soc.*, **15**, 387.

GREEN, G. M. & KASS, E. H. (1964) The Role of the Alveolar Macrophage in the Clearance of Bacteria from the Lung. *J. exp. Med.*, **119**, 167.

GUDEWICZ, P. W., SABA, T. M. & COULSTON, F. (1976*a*) Phagocytic and Bactericidal Activities of Pulmonary Macrophages following Sub-lethal Traumatic Shock. *Proc. Soc. exp. Biol. Med.*, **153**, 262.

GUDEWICZ, P. W., SABA, T. M. & COULSTON, F. (1976*b*) Phagocytic and Metabolic Parameters of Alveolar Macrophages after Sub-lethal Traumatic Shock. *Circ. Shock*, **3**, 337.

HANNA, M. G., ZBAR, B. & RAPP, H. J. (1972) Histopathology of Tumor Regression after Intralesional Injection of *Mycobacterium bovis*. I. Tumor Growth and Metastasis. *J. natn. Cancer Inst.*, **48**, 1441.

HIBBS, J. B., LAMBERT, L. G. & REMINGTON, J. S. (1972) Macrophage Mediated Non-specific Cytotoxicity: Possible Role in Tumor Resistance. *Nature, New Biol.*, **235**, 48.

KAMPSCHMIDT, R. F. & CLABAUGH, W. A. (1964) Effect of Jensen Sarcoma upon the Reticuloendothelial System of Rats of Different Ages. *Proc. Soc. exp. Biol. Med.*, **115**, 681.

KELLER, R. (1973) Cytostatic Elimination of Syngeneic Rat Tumor Cells *In vitro* by Non-specifically Activated Macrophages. *J. exp. Med.*, **138**, 625.

LEVY, M. H. & WHEELOCK, E. F. (1974) The Role of Macrophages in Defence against Neoplastic Disease. *Adv. Cancer Res.*, **20**, 131.

MCKNEALLY, M. F., MAVER, C. & KAUSEL, H. W. (1976) Regional Immunotherapy for Lung Cancer with Intrapleural BCG. *Lancet*, i, 377.

MEGIRIAN, R., SABA, T. M. & STEPHENSON, J. B. (1976) Bacillus Calmette-Guerin Induced Macrophage Stimulation: Humoral Aspects. *J. Reticuloendothel. Soc.*, **20**, 341.

OLD, L. J., BENACERRAF, B., CLARKE, D. H., CARSWELL, E. A. & STOCKERT, E. (1961) The Role of the Reticuloendothelial System in the Host Reaction to Neoplasia. *Cancer Res.*, **21**, 1281.

OUCHI, E., SELVARAJ, R. J. & SBARRA, A. J. (1965) The Biochemical Activities of the Rabbit Alveolar Macrophages During Phagocytosis. *Expl Cell Res.*, **40**, 45.

PIKE, M. C. & SNYDERMAN, R. (1976) Depression of Macrophage Function by a Factor Produced by Neoplasma: A Mechanism for Abrogation of Immune Surveillance. *J. Immunol.*, **117**, 1243.

REYNOLDS, H. Y., KAZMIEROWSKI, J. A. & NEWBALL, H. H. (1975) Specificity of Opsonic Antibodies to Enhance Phagocytosis of *Pseudomonas aeruginosa* by Human Alveolar Macrophages. *J. clin. Invest.*, **56**, 376.

SABA, T. M. & ANTIKATZIDES, T. G. (1975) Humoral Mediated Macrophage Response during Tumour Growth. *Br. J. Cancer*, **32**, 471.

SABA, T. M. & ANTIKATZIDES, T. G. (1976) Decreased Resistance to Intravenous Tumour Cell Challenge during Reticuloendothelial Depression following Surgery. *Br. J. Cancer*, **34**, 381.

SADLER, T. E. & ALEXANDER, P. (1976) Trapping and Destruction of Blood-borne Syngeneic Leukemia Cells in Lung, Liver and Spleen of Normal and Leukemic Rats. *Br. J. Cancer*, **33**, 512.

STERN, K. (1960) The Reticuloendothelial System and Neoplasia. In *Reticuloendothelial Structure and Function*. Ed. Heller, J. H. New York: Ronald Press Co.

VAN DEN BRENK, H. A. S. & KELLY, H. (1974) Potentiating Effect of Prior Local Irradiation of the Lungs on Pulmonary Metastases. *Br. J. Radiol.*, **47**, 332.

VAN DEN BRENK, H. A. S., MOORE, V. & SHARPINGTON, C. (1971) Growth of Metastases from P-388 Sarcoma in the Rat following Whole-body Irradiation. *Br. J. Cancer*, **25**, 186.

VAN DEN BRENK, H. A. S., BURCH, W. M., ORTON, C. & SHARPINGTON, C. (1973) Stimulation of Clonogeneic Growth of Tumour Cells and Metastases in the Lungs by Local X-irradiation. *Br. J. Cancer*, **27**, 291.

VAN DEN BRENK, H. A. S., STONE, M., KELLY, H., ORTON, C. & SHARPINGTON, C. (1974) Promotion of Growth of Tumour Cells in Acutely Inflamed Tissues. *Br. J. Cancer*, **30**, 246.

VAN DEN BRENK, H. A. S., STONE, M. G., KELLY, H. & SHARPINGTON, C. (1976) Lowering of Innate Resistance of the Lungs to the Growth of Blood-borne Cancer Cells in States of Topical and Systemic Stress. *Br. J. Cancer*, **33**, 60.

VAN PUTTEN, L. M., KRAM, L. K. J., VAN DIERENDONCK, H. H. C., SMINK, T. & FUZY, M. (1975) Enhancement by Drugs of Metastatic Lung Nodule Formation after Intravenous Tumor Cell Injection. *Int. J. Cancer*, **15**, 588.